# Supplementary material for: Localization and interaction of interlayer excitons in MoSe2/WSe2 heterobilayers
Source: Nat Commun. 2023 Oct 30;14:6910. doi: 10.1038/s41467-023-42710-8 (PMC10616232; doi:10.1038/s41467-023-42710-8)
Supplement: Supplementary file 1 — Supplementary Information [file 41467_2023_42710_MOESM1_ESM.pdf]

Supplementary Information for: Localization and interaction of interlayer excitons in $MoSe_2/WSe_2$ heterobilayers

Hanlin Fang[1*], Qiaoling Lin[2], Yi Zhang[3], Joshua Thompson[4], Sanshui Xiao[2], Zhipei Sun[3], Ermin Malic[4], Saroj P. Dash[1] and Witlef Wieczorek[1*]

[1]Department of Microtechnology and Nanoscience (MC2), Chalmers University of Technology, 41296, Gothenburg, Sweden.
[2]Department of Electrical and Photonics Engineering, Technical University of Denmark, DK-2800, Kongens Lyngby, Denmark.
[3]Department of Electronics and Nanoengineering and QTF Centre of Excellence, Aalto University, Espoo, 02150, Finland.
[4]Department of Physics, Philipps-Universität Marburg, 35037, Marburg, Germany.

*Corresponding author(s). E-mail(s): hanlin.fang@chalmers.se; witlef.wieczorek@chalmers.se;

Supplementary Text

Supplementary Note 1. Estimation of the depth of potential traps

The temperature-dependent PL intensity change can be described by the Arrhenius equation $I = I_0[1 + a \exp(-E_A/k_B T)]^{-1}$ [1], where E_A is the thermal activation energy and a is a coefficient that is related to the quantum yield of the host material [2]. Through fitting, we obtain an E_A of ∼4 meV for the shallow type-i potential traps and E_A of ∼27 meV for the type-ii moiré potential. In our work, the extracted value of a is about 160, meaning that the non-radiative lifetime is much longer than the radiative lifetime. The reason could be that the strong confinement of excitons reduces the possibility of carriers captured by non-radiative centers [3].

Supplementary Note 2. Calculation of moiré period

The moiré period a_M can be calculated via [4]:

$$a_M = \frac{a_{\mathrm{MoSe_2}} a_{\mathrm{WSe_2}}}{\sqrt{a^2_{\mathrm{MoSe_2}} + a^2_{\mathrm{WSe_2}} - 2a_{\mathrm{MoSe_2}} a_{\mathrm{WSe_2}} \cos\theta}},$$

where $a_{\mathrm{MoSe_2}}$ ($a_{\mathrm{WSe_2}}$) is the lattice constant of monolayer $MoSe_2$ (WSe_2). Considering $a_{\mathrm{MoSe_2}}$($a_{\mathrm{WSe_2}}$)=0.3288 nm (0.3280 nm) [5] and the twist angle (∼58°) of the sample in the main text, we derive a moiré period of ∼9.4 nm.° to 30° due to the symmetry of the crystal [6]. Therefore, a 58° twist angle corresponds to a relative angle of 2° between the layers, as is used in the calculation. A twist angle larger than 30° indicates the stacking type of the heterobilayer, which is H-type in our case.

Supplementary Figures

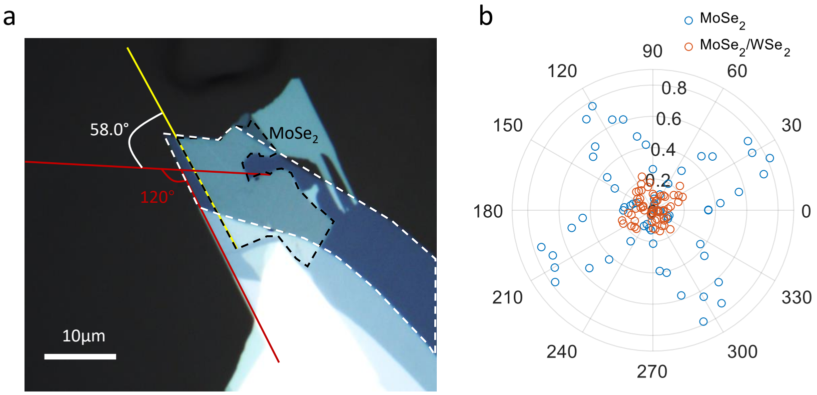

Fig. S1 Characterization of twist angle. (a) Optical image of the stacked $MoSe_2/WSe_2$ heterobilayer on PDMS. The twist angle is measured between the straight edges of the monolayers and determined to be $58.0° \pm 0.6°$, where the uncertainty is given by the range of twist angles due to the pixelated edges of the image. Scale bar: 10 μm. (b) Polarization-resolved SHG measurement. The SHG intensity of the heterobilayer is remarkably weak compared to monolayer $MoSe_2$, confirming that the stack type is H-stack [7].

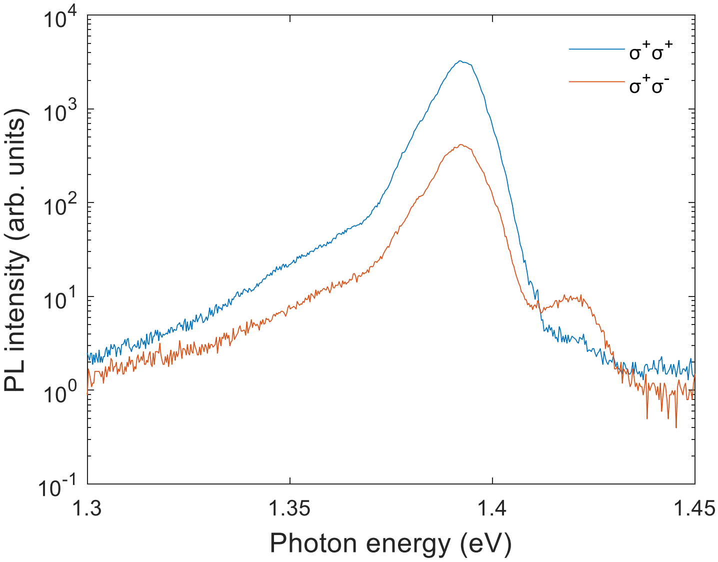

Fig. S2 Valley polarization of S and T with a pump power of 66 μW at 4.9 K.

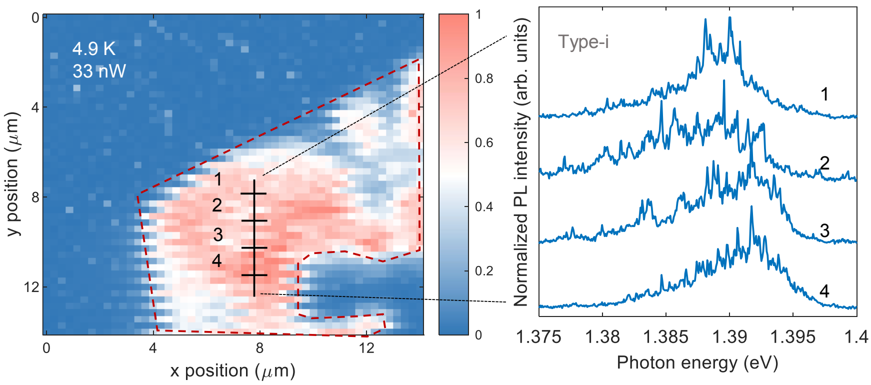

Fig. S3 Spatial distribution of type-i IXs. PL map obtained by integrating the PL spectrum between ∼1.333 and ∼1.425 eV at 4.9 K. The sharp emission lines (denoted as type-i) from the marked positions are shown in the right panel, which is ascribed to defect-bound excitons.

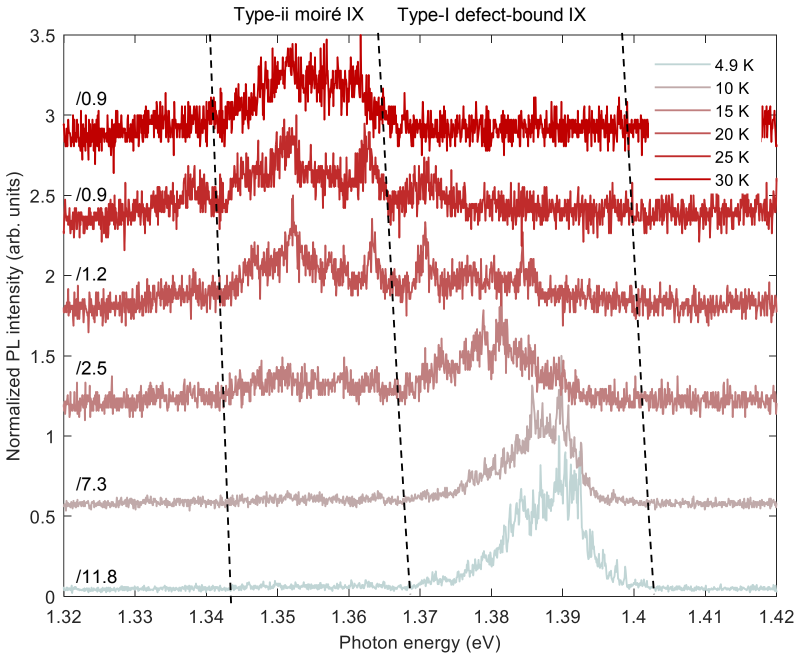

Fig. S4 PL emission as a function of temperature with a pump power of 33 nW. Each spectrum is normalized with its own peak intensity.

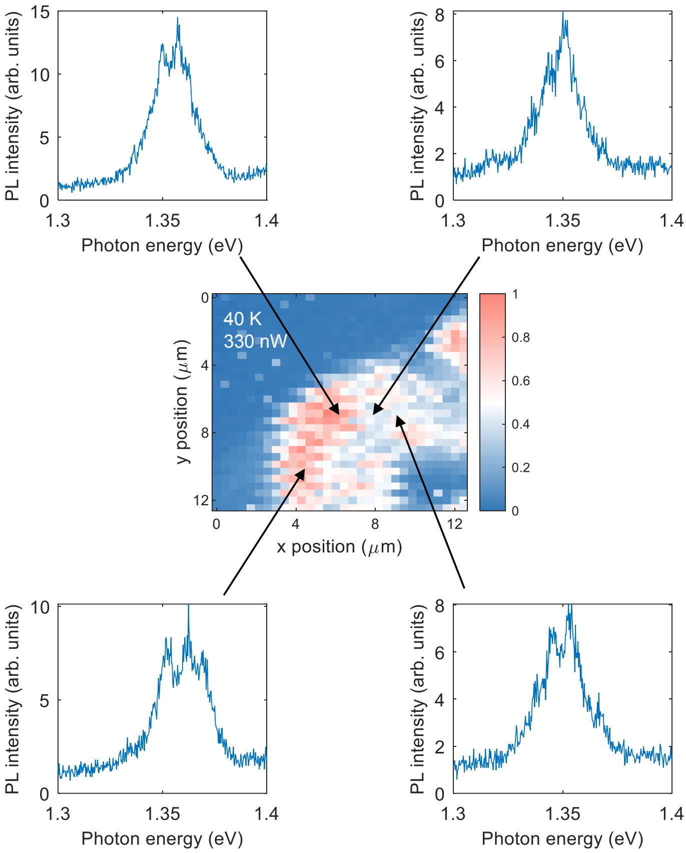

Fig. S5 PL map of type-ii localized IXs with a pump power of 330 nW at 40 K. The multipeak feature can be observed from various positions, however, the energy spacing between adjacent peaks varies with position.

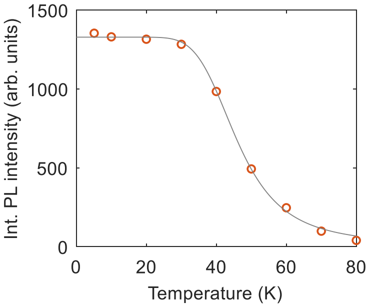

Fig. S6 Estimation of the depth of the type-ii potential via fitting by the Arrhenius equation. Note that the data is acquired from a different position than the inset shown in Fig. 2d of the main text.

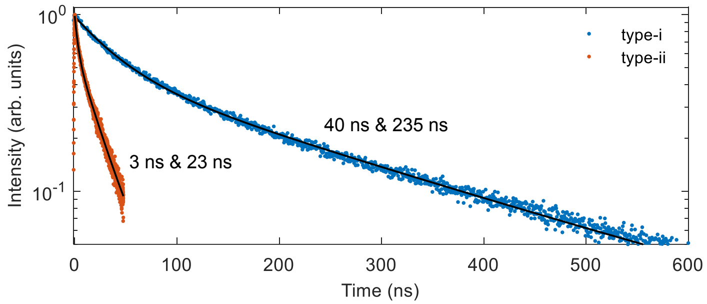

Fig. S7 Time-resolved PL dynamics of type-i and type-ii IXs. The solid lines represent the biexponential fits to the data, yielding the lifetime of type-i (∼40 ns and ∼235 ns) and type-ii (∼3 ns and ∼23 ns) IX. The lifetime of type-i (ii) localized IXs is measured at 4.9 K (40 K). Note that the measured type-i and type-ii IX lifetime is similar to the reported lifetime of sharp emission lines [8] and moiré-related peaks [9], respectively.

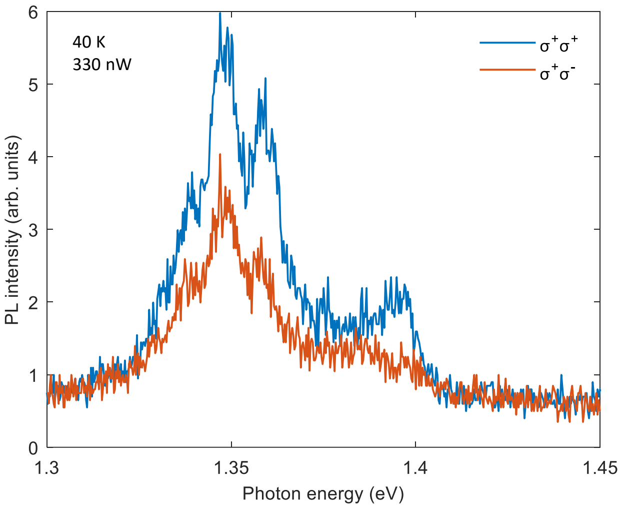

Fig. S8 Valley polarization of type-ii localized IXs with a pump power of 330 nW.

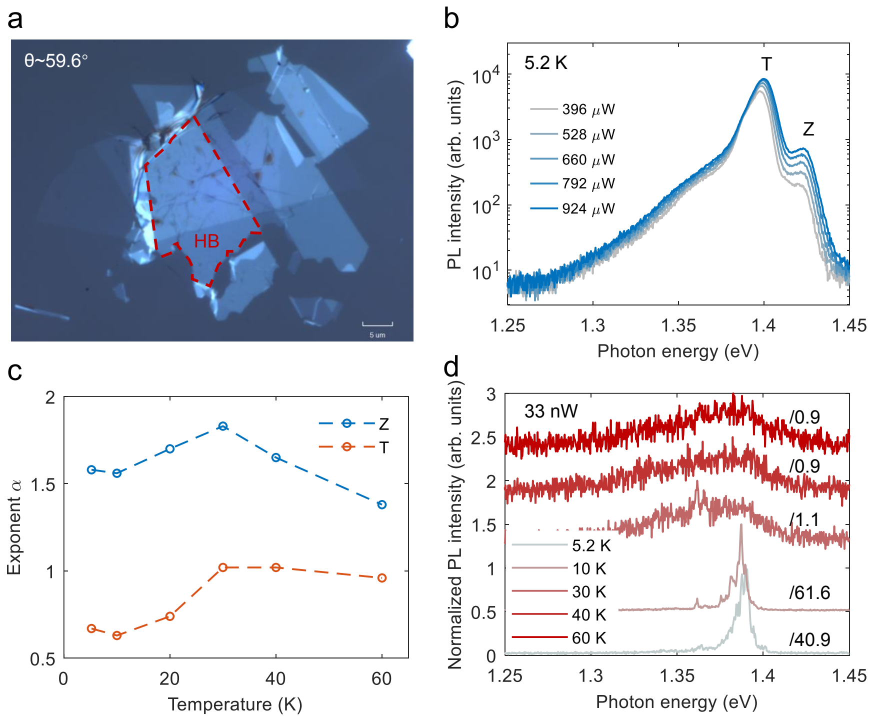

Fig. S9 Reproducibility of the temperature-dependent PL emission behavior in another sample. (a) Optical image of the hBN-encapsulated $MoSe_2/WSe_2$ heterobilayer. (b) Power-dependent PL spectrum in the high excitation regime. (c) Temperature-dependent change of exponent α. (d) Evolution of PL spectrum with temperature. Each spectrum is normalized with its peak intensity. No clear type-ii emissions emerge with increasing temperature, which is possibly due to the atomic reconstruction for the small twisted ($< 1^\circ$) heterobilayer [10].

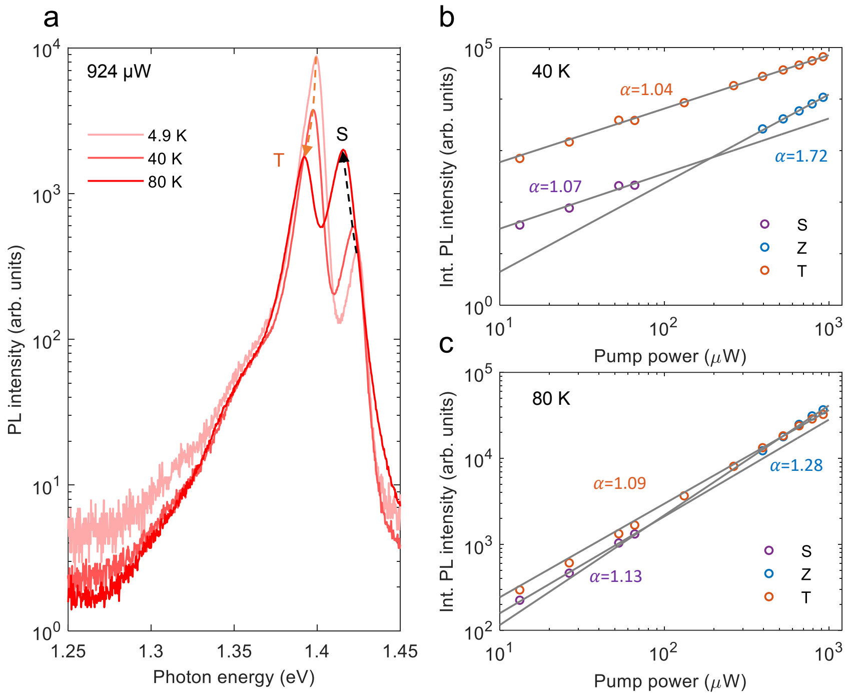

Fig. S10 Power dependence of PL emission at different temperatures. (a) Evolution of PL spectrum with a pump power of 924 μW. With increasing temperature to 80 K, the IXX gradually vanishes (see Fig. 4c of the main text) and the S-IX takes over the emission peak. The increasing (decreasing) PL intensity of S(T)-IX with temperature is consistent with previous work [11]. (b) and (c) show the integrated PL intensity as a function of pump power at 40 K and 80 K, respectively. We attribute the predominant emission at the different temperatures to the Z (high excitation regime) or S peak (low excitation regime) by performing power-dependent measurements. We use a power law to fit the data and extract the exponent α, as shown in (b) and (c). With increasing temperature, we gradually do not see a difference of α between low and high excitation regimes and its value is approaching 1 (also see Fig. 4c in the main text).

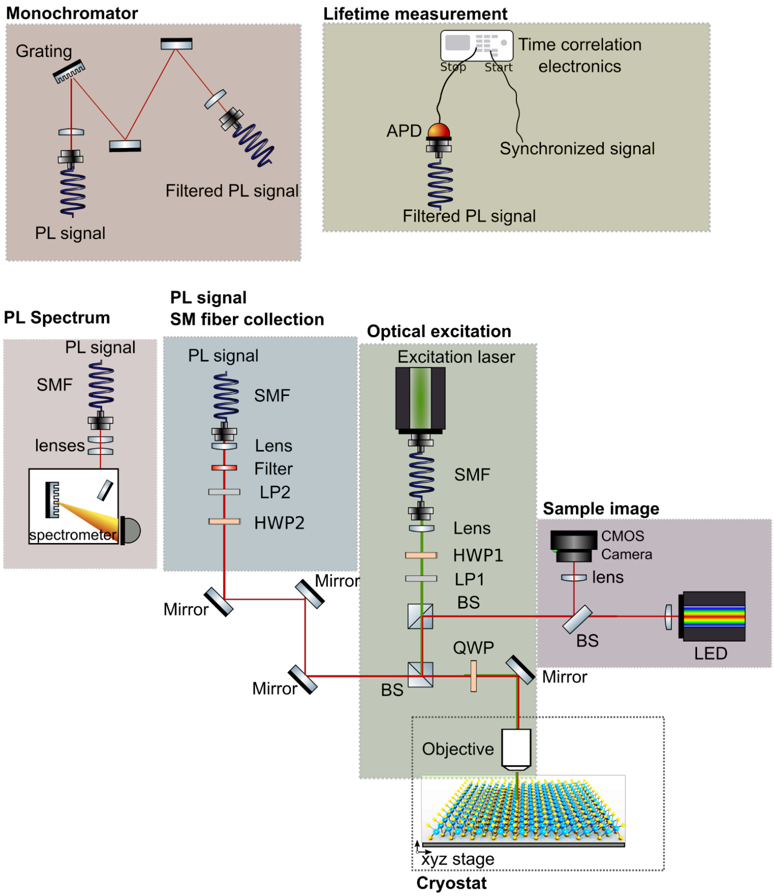

Fig. S11 Schematics of the PL measurement setup. LP: linear polarizer. HWP: half-waveplate. BS: beam splitter. SMF: single-mode fiber. The PL signal is sent to the spectrometer for measuring the PL spectrum, to the monochromator to filter the desired emission peak and measure exciton lifetime. LP1 is set to be horizontally linear polarized. LP2 and QWP are only used for valley polarization measurements. QWP is used to convert the linearly polarized laser emission to circularly polarized and then excite the sample. The circularly-polarized PL signal will be converted into a linear polarized signal, which is analyzed by LP through rotating HWP2. QWP and LP2 are removed when performing power- and temperature-dependent PL spectrum, and lifetime measurements.

References

[1] Luo, Y., Liu, N., Li, X., Hone, J. C. & Strauf, S. Single photon emission in WSe_2 up 160 K by quantum yield control. 2D Materials **6**, 035017 (2019).

[2] Leroux, M. et al. Temperature quenching of photoluminescence intensities in undoped and doped GaN. Journal of Applied Physics **86**, 3721–3728 (1999).

[3] Amani, M. et al. Near-unity photoluminescence quantum yield in mos2. Science **350**, 1065–1068 (2015).

[4] Zhang, N. et al. Moiré intralayer excitons in a mose2/mos2 heterostructure. Nano letters **18**, 7651–7657 (2018).

[5] Huang, C. et al. Lateral heterojunctions within monolayer mose 2–wse 2 semiconductors. Nature materials **13**, 1096–1101 (2014).

[6] Rivera, P. et al. Interlayer valley excitons in heterobilayers of transition metal dichalcogenides. Nature nanotechnology **13**, 1004–1015 (2018).

[7] Hsu, W.-T. et al. Second harmonic generation from artificially stacked transition metal dichalcogenide twisted bilayers. ACS nano **8**, 2951–2958 (2014).

[8] Seyler, K. L. et al. Signatures of moiré-trapped valley excitons in $MoSe_2/WSe_2$ heterobilayers. Nature **567**, 66–70 (2019).

[9] Tran, K. et al. Evidence for moiré excitons in van der Waals heterostructures. Nature **567**, 71–75 (2019).

[10] Rosenberger, M. R. et al. Twist angle-dependent atomic reconstruction and moiré patterns in transition metal dichalcogenide heterostructures. ACS nano **14**, 4550–4558 (2020).

[11] Zhang, L. et al. Highly valley-polarized singlet and triplet interlayer excitons in van der Waals heterostructure. Physical Review B **100**, 041402 (2019).
